# Mental ill-health across the continuum of body mass index

**DOI:** 10.1186/1471-2458-11-765

**Published:** 2011-10-05

**Authors:** Shona J Kelly, Mark Daniel, Eleonora Dal Grande, Anne Taylor

**Affiliations:** 1 Social Epidemiology and Evaluation Group, Sansom Institute for Health Research, University of South Australia, Australia; 2 Department of Medicine, St Vincent's Hospital, The University of Melbourne, Australia; 3 Population Research & Outcome Studies (PROS), Discipline of Medicine, Faculty of Health Science, The University of Adelaide, Australia

**Keywords:** BMI, non-linear associations, underweight, mental health, health behaviours, obese

## Abstract

**Background:**

Several studies have found a non-linear relationship between mental ill-health and BMI with higher rates in both the underweight and the obese. This study evaluated the shape of the relationship between BMI and distress, suicidal ideation and self-reported mental ill-health conditions in a large population sample.

**Methods:**

Data were drawn from the South Australian Monitoring and Surveillance System (SAMSS) for the years 2002 to 2009 (n = 46,704). SAMSS monitors population trends in state and national risk factors and chronic diseases. Samples are drawn from all households with a functioning number in the Australian White Pages. Computer assisted telephone interviews collected information on self-reported height and weight, demographic and health behaviours. Respondents completed the Kessler Distress and suicidal ideation scales and reported specific mental ill-health conditions. BMI was categorized into deciles to allow for assessment of the shape of any associations with other variables. Logistic regression was used to examine associations between each mental ill-health condition and BMI-decile controlling for age in the base model. This was followed by a full model that added SES and the health-adverse coping behaviours of smoking, alcohol and physical activity to test for changes from the base model.

**Results:**

Non-linear associations were observed between BMI-decile and mental ill-health but statistically significantly greater odds of mental ill-health were observed only in the obese and not in the underweight after controlling for age, health-adverse behaviours and socioeconomic status. The association between BMI and mental ill-health might best be described as 'threshold'. Elevated odds were apparent for middle-aged persons, whereas younger and older individuals had a significantly lower odds of having a mental ill-health condition.

**Conclusions:**

In conclusion, this study has provided no support for the hypothesis of increased mental ill-health problems in the underweight but it has demonstrated the non-linear relationships between BMI and mental ill-health and between BMI and health-adverse behaviours. Non-linear relationships with BMI need to be recognized and addressed during analysis.

## Background

In a recent analysis of data from the Health Survey for England [1] of factors which might be associated with the higher mortality rates often found in the underweight body mass index (BMI) category [2-8], the study found that the underweight were younger, poorer, less active, more likely to smoke, and twice as likely to report mental ill-health problems than those in the acceptable-weight BMI category (6.8% vs. 3.3% (p < .05), respectively) [1]. While this report was specific to the understudied, underweight individuals, most research on mental ill-health and weight has instead targeted overweight and obese individuals. Studies of mental ill-health among overweight or obese persons have generally [9] but not always [10] observed a greater frequency of mental ill-health problems.

It is well established that BMI has a U- or J-shaped association with mortality (e.g., [6,7]). The health, social and cultural factors associated with obesity are well characterised but few studies have assessed these and related associations for underweight persons. Little evidence exists to explain why the underweight have a greater risk of dying [2-8]. Many authors have suggested that underweight persons evidencing greater mortality have undiagnosed disease[11]. But previous work by one of our team found no evidence for this undiagnosed disease in underweight persons although, underweight persons did have a greater prevalence of smoking and low physical activity [1]. These health behaviour findings are similar to those reported by others [6,12-14]. The underweight, compared to those in the acceptable-weight category, are also more likely to have the most 'healthy' levels of biochemical risk factors for cardiovascular disease [1,6,15,16] and less occluded coronary arteries [17] which is consistent with their low rates of cardiometabolic disease.

Mental ill-health is a possible explanation for the higher mortality in the underweight. Social factors associated with mental ill-health conditions and greater risks of mortality are more common in the underweight than those of normal weight. For example, compared to those of normal weight the underweight persons are more likely to be unmarried [13,15], unemployed [18] or on disability pension [19]. Outside of cardiovascular disease many health conditions also have a J- or U-shaped association with BMI, namely: depression [16,20], disability and arthritis [21], and self-reported health [13,15,18,22]. Self-reported health status has been shown to have a significant contribution from mental ill-health as well as physical health [23] suggesting that some of the increased mortality for the underweight may be due to mental ill-health problems, perhaps acting through health behaviours. Mental ill-health problems were identified as a significant reason for being on a disability pension in a systematic review of the relationship between BMI and the risk of being on a disability pension [19] and in this study the authors found a J-shaped relationship with BMI. They also identified a greater risk of mortality in the underweight was seen for alcohol abuse and mental ill-health disorders [19]. Such observations support the view that the underweight have more mental ill-health problems.

Health problems at the bottom on the BMI-continuum in developed countries have been difficult to identify in published research as repeatedly, in the research which identifies J- or U-shaped associations, the underweight are excluded from analysis because the focus is on overweight and obesity only (eg. [24]). In some cases, if the underweight are included in the analysis, any statistically significant greater risks for them are essentially ignored (eg. [25]). This oversight has led to a false view that BMI is associated in a linear fashion with health (i.e., decreasing BMI equals improving health), when the reality is curvilinear for most health conditions and risk factors except for cardiometabolic disease.

With the exception of the mental ill-health of those with eating disorders (eg., [26]), only a few studies report on the mental ill-health of the underweight as well as those with excess weight. Studying mental ill-health in low BMI persons with eating disorders might not be representative of the entire population of persons with low BMI. Calculating the prevalence of distress using a paper on the Australian Health Survey[27], we noted that a high score on the Kessler Psychological Distress Scale was much more common in the underweight than other BMI categories: 9.9% in underweight vs. 3.4%, 3.4%, 4.9% in those of acceptable weight, the overweight and the obese, respectively (calculated from Table 1[27]). A survey of active US Military personnel found that underweight males had twice the prevalence of depressive symptoms as compared to all the other BMI categories [20]. However, for women only the obese had a significantly greater prevalence of depressive symptoms after accounting for demographic factors and health-adverse/coping behaviours. Research on elderly persons with depression found that those underweight or obese were more likely to be depressed than those of normal weight or overweight [28]. Further, a population-based survey in Canada of residents aged 18-64 years did not find an elevated risk for anxiety or depression in underweight women. This study did observe, however, a greater odds of anxiety or depression in underweight relative to acceptable weight men (OR = 5.9, 95% CI 1.6, 21.6) [29]. Using non-linear modelling techniques two recent publications have also identified a u-shaped distribution between BMI categories and depression [30,31] and noted different prevalences for the categories between the genders. While too few studies exist from which to draw firm conclusions, research conducted thus far suggests that there may be significant gender differences and it is likely that age may also play a role in the association between BMI and adverse mental ill-health status.

**Table 1:** Descriptive characteristics of study participants according to gender and BMI-decile

	Males										Females									
	BMI Decile										BMI Decile									
	UW%	Ac	Ac	Ac	Ac	Ow	Ow	Ow	Ob	Ob	U%	Ac	Ac	Ac	Ac	Ow	Ow	Ow	Ob	Ob
	1	2	3	4	5	6	7	8	9	10	1	2	3	4	5	6	7	8	9	10
**% of gender**	5.3	7.9	9.3	10.7	11.4	12.6	11.6	12.3	10.6	8.4	13.5	11.6	10.5	9.6	9.1	8	8.8	8.2	9.6	11.4
**age group**																				
**18-39 (%)**	38.4	32.2	27.0	23.9	20.5	19.9	18.0	17.0	16.1	16.0	32.5	26.0	22.9	19.9	17.5	16.9	13.6	14.5	14	15.1
**40-59 (%)**	39.9	39.3	41.0	42.3	41.7	41.6	42.8	39.9	39.2	32.4	38.1	38.1	40.8	46.4	45.0	46.8	48.0	46.3	45.2	38.5
**60+ (%)**	21.7	28.5	32.0	33.8	37.8	38.6	39.2	43.1	44.7	51.6	29.3	35.9	36.2	33.7	37.5	36.3	38.4	39.2	40.8	46.4
																				
**smoking**																				
**non-smoker (%)**	36.2	35.0	37.3	35.3	33.5	34.3	31.8	32.4	30.6	29.7	49.7	49.9	51.3	50.9	51.2	49.4	50.4	51.5	49.8	46.7
**ex-smoker (%)**	37.2	40.5	42.2	45.3	49.5	49.9	50.4	51.5	52.6	52.1	31.8	34.5	33.9	37.3	35.6	37.1	36.6	36.5	37.4	40.5
**smoker (%)**	26.5	24.5	20.5	19.4	17.0	15.8	17.8	16.1	16.8	18.3	18.5	15.6	14.8	11.8	13.2	13.5	13.0	11.9	12.8	12.8
																				
**alcohol consumption**																				
**abstainer (%)**	18.2	14.5	12.6	12.5	11.7	11.5	10.8	12.0	13.0	16.7	26.2	21.8	22.5	23.1	23.8	24.6	24.3	26.8	28.9	32.5
**low risk (%)**	48.2	45.0	45.3	45.8	46.8	48.2	48.3	49.1	51.0	51.7	49.0	48.3	49.2	50.9	51.6	51.9	53.7	53.4	54.6	54.4
**risky (%)**	31.2	37.3	40.0	39.7	40.0	39.3	39.0	36.9	33.6	28.5	23.1	21.1	26.6	24.9	23.5	21.8	20.6	18.0	15.5	11.4
**high risk (%)**	2.5	3.2	2.2	2.0	1.5	1.0	1.9	2.0	2.4	3.2	1.7	1.7	1.8	1.1	1.1	1.7	1.5	1.7	1.1	1.7
																				
**physical activity**																				
**none (%)**	22.1	17.5	16.7	18.0	18.4	19.3	21.6	20.7	23.9	29.7	20.5	17.6	17.3	19.3	19.2	21.3	23.1	21.8	25.2	29.4
**insufficient (%)**	28.7	27.5	24.1	24.2	24.5	26.7	27.2	28.5	31.7	31.8	31.8	28.7	31.5	31.5	32.6	32.6	34.3	33.6	35.7	39.4
**sufficient (%)**	49.2	55.0	59.2	57.9	57.1	54.0	51.2	50.8	44.4	38.5	47.7	53.7	51.2	49.2	48.2	46.1	42.6	44.7	39.1	31.2
																				
**education**																				
**<= high school (%)**	57.5	52.0	48.6	46.3	47.3	46.6	48.5	50.3	52.8	53.2	60.0	57.4	60.8	61.9	62.5	63.4	66.3	65.5	66.7	67.4
**trade/cert./dipl. (%)**	23.4	25.7	27.0	31.1	29.5	31.7	31.9	32.4	32.2	32.2	18.0	19.1	18.5	18.7	19.4	18.6	18.5	18.0	19.1	18.9
**degree + (%)**	19.1	22.3	24.4	22.5	23.2	21.7	19.6	17.3	15.0	14.6	22.0	23.5	20.7	19.4	18.1	17.9	15.2	16.4	14.2	13.8
																				
income '000 AUD*	3 (3)	4 (2)	4(2)	4 (2)	4 (3)	4 (3)	4 (3)	4 (3)	4 (3)	4 (2)	3 (3)	4 (3)	3 (3)	3 (3)	3 (3)	3 (3)	3 (3)	3 (3)	3 (3)	3 (2)

* median and IQR

"% UW = underweight, Ac = acceptable weight, Ow = overweight, Ob = obese"

The shape of the relationship between BMI-deciles and the prevalence of distress, suicidal ideation and self-reported mental ill-health conditions was examined by comparing, for each gender separately, the age adjusted prevalence across the deciles. Also, the impact of potential confounders such as health-adverse behaviours, or socioeconomic status (SES), was examined to test their affect on the relationship between BMI and the mental ill-health measures.

## Methods

Data were drawn from the South Australian Monitoring and Surveillance System (SAMSS) [32] for the years 2002 to 2009 (n = 46,704). SAMSS was established in 2002 by the Population Research and Outcome Studies Unit at SA Health in South Australia. SAMSS monitors population trends in state and national risk factors and chronic diseases for the Department of Health. Ethical approval was granted by the University of South Australia Human Research Ethics Committee (#0000020364).

All households in South Australia with a functioning telephone and telephone number listed in the Australian Electronic White Pages are eligible for selection into the sample. Interviews are conducted by telephone each month for approximately 600 randomly selected people. Using Computer Assisted Telephone Interview technology, the SAMSS questionnaire collects information on a wide variety of health-related data. Measures relevant to this analysis include: self-reported height and weight (from which BMI was calculated), demographic and health behaviours information, and measures of mental well-being.

For this study, from the initial sample of telephone numbers (n = 96265), 18597 were excluded for a variety of reasons: telephone number non-connected, non-residential telephone number, fax or modem lines, or household previously selected. From the remaining 77668 eligible numbers, 52605 interviews were conducted with the householder who had the next birth date. No substitutions were made if this person was not available leading to an overall response rate of 67.7%. Non-response was due to refusal (14.2%), contact not being established after six attempts (9.4%), incapacitated (i.e., illness) (3.3%), respondent speaking only a language other than English (2.5%), respondent unavailable (2.7%), and termination of the interview by the respondent (0.2%). The final study sample was 43,214 participants after restricting the sample to those with complete data on BMI and the mental ill-health scales (not all scales were asked each month due to cost and length of interview). BMI was restricted to values within 5 standard deviations of the mean as some of the extreme outliers (n = 407) did not seem plausible with a final sample of 42,807.

As the relationships under examination have been shown to be non-linear, BMI, alcohol consumption and age group were converted into categorical variables for analysis. BMI was categorized into deciles based on the study sample with upper cut-offs for each decile of 20.90, 22.45, 23.71, 24.80, 25.89, 27.01, 28.39, 30.12, 33.01, with the highest value of 52.72. The lowest decile corresponds roughly to the WHO-defined underweight BMI category (BMI = <18.5), the 9^th ^and 10^th ^to the WHO-defined obese (BMI = ≥30) and the 6^th^, 7^th ^and 8^th ^to the WHO-defined overweight BMI category (BMI = 25.0-29.9. The remaining deciles roughly encompass the WHO-defined acceptable weight category (BMI = 18.5-24.9). Decile 4, >23.71 and < = 24.80 kg/m^2^, was the reference category for analyses.

Distress was measured with the Kessler Psychological Distress Scale-10 [33]. Respondents are asked to select from "none", "a little", "some", "most" or "all" "of the time" to each of 10 questions. Examples of the questions include: "About how often did you feel hopeless?" or "About how often did you feel that everything is an effort?". Responses are scored from one to five and summed (range 10 to 50). A score greater than 22 was labelled as "distressed"[33]. Four questions on suicidal ideation were drawn from the General Health Questionnaire [34]. Likert-type responses were dichotomised into negative (score = 0) or positive responses (score = 1) with a positive response to any question being considered indicative of "suicidal ideation". The questions have not always been included in the survey so analyses including suicidal ideation have a smaller n. Participants were also asked specific questions about their mental ill-health status including whether they had been told by a doctor that they had 1) anxiety, 2) depression, 3) a stress-related problem, or 4) other mental ill-health problem (all recorded as yes or no). These four questions were also aggregated into a single measure that confirmed one or more positive responses to the four questions. A separate binary variable indicated whether any response concerned a current condition (i.e., within the past year).

Potential confounders considered in analyses include health-adverse behaviours and socioeconomic status (SES). Respondents indicated whether they were a "never smoker", "ex-smoker" or "current smoker". Alcohol consumption (derived from the number of alcoholic drinks per day and the number of times per week alcohol was consumed) and physical activity (derived on the amount of walking and moderate and vigorous activity in a one-week period) were based on national guidelines [35,36]. Alcohol consumption is categorised as "non-drinker", "low risk", "risky, and high risk". Physical activity was classified as "no activity", "insufficient activity" and "sufficient activity". SES was assessed as education and income. Education was categorised as "high school or less", "trade/certificate/diploma", and "university degree or higher". Income was treated as a continuous measure.

### Statistical analysis

Data was imported into PASW Statistics (version 18) for analysis. For each BMI decile the proportion classified as distressed, suicidal, or reporting a doctor-diagnosed mental ill-health condition was calculated and plotted for each gender separately.

Logistic regression was used to examine associations between each mental ill-health condition and BMI-decile controlling for age in the base model. This was followed by a full model that added SES and the health-adverse coping behaviours of smoking, alcohol and physical activity to test for changes from the base model.

## Results

Descriptive characteristics for the study participants, according to BMI decile, are given in Table 1. Large gender differences were apparent for some variables, but for men and women together, the underweight (decile 1) were represented more by the younger age groups and the obese (deciles 9 and 10) were represented more by the oldest age category. In both men and women current smokers were more frequent in the lower BMI-deciles which incorporate the underweight and acceptable weight categories. The prevalence of alcohol abstainers and "risky" drinkers showed u-shaped distributions with BMI-decile such that the underweight and obese are more likely to be abstainers than the intermediate deciles. The relationship between BMI-decile and physical activity was also u-shaped with the underweight and obese less likely to engage in physical activity. Underweight and obese males were less likely to have a university degree and more likely to have less than a high school education. In women there was a gradient of increasing education with decreasing BMI-decile.

**Figure 1:**
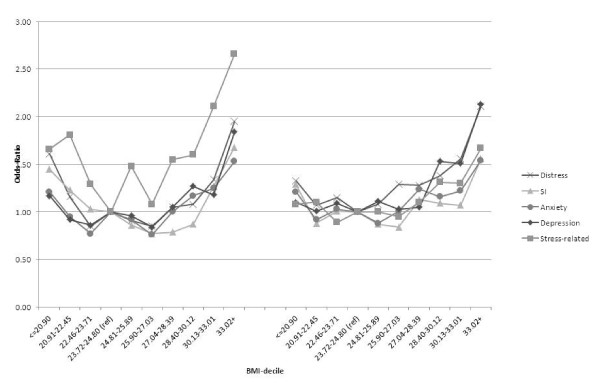
**Age-adjusted odds ratio of mental ill-health**.

Figure 1 shows the age-adjusted odds for mental ill-health conditions by BMI-decile. While the relationships are clearly non-linear there is only a faint suggestion of a J-shaped relationship for most conditions for either gender. The exception is stress-related problems in males but the Ors show considerable variability which is likely due to small numbers. The graphed relationships suggest little difference in the prevalence of mental ill-health conditions for BMI-deciles less than that corresponding to the onset of overweight after which the odds of mental ill-health conditions rises with increasing deciles of BMI.

Tables 2 and 3 present the multivariate logistic regression models for K10-diagnosed distress, a positive suicidal ideation score anxiety, depression, a stress-related problem or 'other' mental ill-health problem for men and women. Rather than report the findings in excruciating detail we refer the reader to the tables and here describe the shape of the distributions between mental ill-health and BMI deciles. The multivariate logistic regression models (model 1), which include BMI-decile and age, confirmed that the lowest BMI-decile differed significantly from decile 4 for distress with the remainder of the significant differences generally concentrated in the top BMI deciles (upper overweight and obese categories) (see Tables 2 and 3). For men and women, the risk for all mental ill-health conditions is greatest in the middle aged (age 40-59) and is significantly lower for both younger and older age groups.

**Table 2:** Multivariate regression of the odds of mental ill-health according to BMI-decile and covariates, OR (p) - Males

	Distress		Suicidal Ideation		Anxiety		Depression		Stress-related		Other	
	basic	full	basic	full	basic	full	basic	full	basic	full	basic	full
**age (overall)**	(<.001)	(<.001)	(.001)	(<.001)	(<.001)	(<.001)	(<.001)	(<.001)	(<.001)	(<.001)	(<.001)	(<.001)
**age 18-39**	0.85 (.027)	1.07 (.449)	0.77 (.030)	0.88 (.328)	0.80 (.020)	0.88 (.244)	0.74 (<.001)	0.82 (.052)	0.67 (<.001)	0.69 (.001)	0.91 (.568)	1.12 (.557)
**age 40-59 (ref)**	1.00	1.00	1.00	1.00	1.00	1.00	1.00	1.00	1.00	1.00	1.00	1.00
**age 60+**	0.69 (<.001)	0.42 (<.001)	0.66 (<.001)	0.42 (<.001)	0.67 (<.001)	0.48 (<.001)	0.66 (<.001)	0.40 (<.001)	0.50 (<.001)	0.42 (<.001)	0.34(<.001)	0.16 (<.001)
**BMI decile (overall)**	(<.001)	(<.001)	(<.001)	.031	(<.001)	(.011)	(<.001)	(<.001)	(<.001)	(<.001)	(<.001)	(.011)
**< = 20.90**	1.61 (<.001)	1.11 (.530)	1.45 (.083)	1.08 (.771)	1.21 (.306)	0.86 (.474)	1.17 (.358)	0.74 (.137)	1.66 (.016)	1.29 (.326)	1.90 (.088)	0.92 (.876)
**20.91-22.45**	1.16 (.250)	1.07 (.676)	1.23 (.296)	1.28 (.265)	0.95 (.758)	0.88 (.500)	0.92 (.598)	0.84 (.305)	1.81 (.001)	1.93 (.002)	1.78 (.094)	1.96 (.096)
**22.46-23.71**	0.85 (.224)	0.77 (.104)	1.03 (.877)	1.06 (.797)	0.77 (.133)	0.63 (.022)	0.86 (.321)	0.70 (.038)	1.29 (.181)	1.33 (.193)	1.22 (.590)	1.36 (.475)
**23.72-24.80 (ref)**	1.00	1.00	1.00	1.00	1.00	1.00	1.00	1.00	1.00	1.00	1.00	1.00
**24.81-25.89**	0.91 (.466)	0.97 (.814)	0.86 (.442)	0.97 (.905)	0.92 (.578)	0.86 (.383)	0.96 (.775)	0.93 (.659)	1.48 (.026)	1.60 (.020)	1.37 (.351)	2.00 (.085)
**25.90-27.03**	0.85 (.173)	0.94 (.693)	0.77 (.168)	0.95 (.822)	0.76 (.084)	0.67 (.031)	0.84 (.218)	0.87 (.359)	1.08 (.659)	1.23 (.315)	0.83 (.620)	1.27 (.574)
**27.04-28.39**	1.05 (.697)	1.07 (.644)	0.79 (.229)	0.95 (.799)	1.00 (.989)	0.96 (.805)	1.05 (.737)	1.04 (.797)	1.55 (.012)	1.80 (.003)	0.98 (.956)	1.20 (.677)
**28.40-30.12**	1.08 (.494)	1.22 (.135)	0.87 (.442)	0.96 (.866)	1.17 (.279)	1.10 (.554)	1.27 (.065)	1.21 (.194)	1.60 (.006)	1.79 (.003)	1.76 (.075)	2.33 (.026)
**30.13-33.01**	1.34 (.011)	1.29 (.063)	1.27 (.183)	1.35 (.136)	1.25 (.144)	1.16 (.382)	1.18 (.215)	1.13 (.414)	2.11 (<.001)	2.19 (<.001)	2.35 (.006)	2.94 (.004)
**33.02+**	1.95 (<.001)	1.64 (<.001)	1.68 (.004)	1.71 (.007)	1.53 (.005)	1.16 (.400)	1.84 (<.001)	1.46 (.010)	2.66 (<.001)	2.61 (<.001)	3.07 (<.001)	2.69 (.009)
**smoking (overall)**		(<.001)		(<.001)		(<.001)		(<.001)		(.001)		(.004)
**non-smoker (ref)**		1.00		1.00		1.00		1.00		1.00		1.00
**ex-smoker**		1.33 (.001)		1.19 (.148)		1.32 (.008)		1.35 (.001)		1.11 (.301)		1.21 (.354)
**smoker**		1.93 (<.001)		1.73 (<.001)		1.90 (<.001)		1.85 (<.001)		1.53 (<.001)		1.96 (.002)
**alcohol consumption (overall)**		(<.001)		(.001)		(<.001)		(<.001)		(<.001)		(<.001)
**abstainer**		1.60 (<.001)		1.54 (.002)		1.90 (<.001)		1.80 (<.001)		1.50 (.001)		2.02 (<.001)
**low risk (ref)**		1.00		1.00		1.00		1.00		1.00		1.00
**risky**		0.97 (.630)		1.25 (.043)		1.12 (.230)		1.03 (.726)		0.96 (.657)		0.80 (.261)
**high risk**		1.55 (.016)		2.11 (.004)		2.44 (<.001)		1.87 (.002)		1.77 (.011)		0.86 (.760)
**physical activity (overall)**		(<.001)		(.008)		(.083)		(.020)		(.153)		(.718)
**no activity**		1.72 (<.001)		1.44 (.002)		1.13 (.246)		1.26 (.011)		1.15 (.203)		1.04 (.868)
**insufficient**		1.37 (<.001)		1.23 (.069)		1.24 (.027)		1.19 (.037)		1.90 (.065)		1.16 (.425)
**sufficient (ref)**		1.00		1.00		1.00		1.00		1.00		1.00
**Education (overall)**		(.388)		(.002)		(<.001)		(.001)		(.001)		(<.001)
**high school or less**		1.00		1.00		1.00		1.00		1.00		1.00
**"trade, cert, dipl"**		0.91 (.195)		1.04 (.713)		1.07 (.460)		0.90 (.220)		1.34 (.002)		0.82 (.325)
**degree or higher**		0.92 (.419)		1.58 (.001)		1.62 (<.001)		1.36 (.002)		1.43 (.002)		2.08 (<.001)
**income ('000 AUD)**		0.69 (<.001)		0.68 (<.001)		0.77 (<.001)		0.71 (<.001)		0.85 (<.001)		0.55 (<.001)
**N**	18956	14882	10576	9227	18995	14907	18995	14907	18995	14907	18995	14907

**Table 3:** Multivariate regression of the odds of mental ill-health according to BMI-decile and covariates, OR (p) -Females

	Distress		Suicidal Ideation		Anxiety		Depression		Stress-related		Other	
	basic	full	basic	full	basic	full	basic	full	basic	full	basic	full
**age (overall)**	(<.001)	(<.001)	(.001)	(<.001)	(<.001)	(<.001)	(<.001)	(<.001)	(<.001)	(<.001)	(<.001)	(<.001)
**age 18-39**	0.85 (.027)	1.07 (.449)	0.77 (.030)	0.88 (.328)	0.80 (.020)	0.88 (.244)	0.74 (<.001)	0.82 (.052)	0.67 (<.001)	0.69 (.001)	0.91 (.568)	1.12 (.557)
**age 40-59 (ref)**	1.00	1.00	1.00	1.00	1.00	1.00	1.00	1.00	1.00	1.00	1.00	1.00
**age 60+**	0.69 (<.001)	0.42 (<.001)	0.66 (<.001)	0.42 (<.001)	0.67 (<.001)	0.48 (<.001)	0.66 (<.001)	0.40 (<.001)	0.50 (<.001)	0.42 (<.001)	0.34 (<.001)	0.16 (<.001)
**BMI decile (overall)**	(<.001)	(<.001)	(<.001)	.031	(<.001)	.011	(<.001)	(<.001)	(<.001)	(<.001)	(<.001)	(.011)
**< = 20.90**	1.61 (<.001)	1.11 (.530)	1.45 (.083)	1.08 (.771)	1.21 (.306)	0.86 (.474)	1.17 (.358)	0.74 (.137)	1.66 (.016)	1.29 (.326)	1.90 (.088)	0.92 (.876)
**20.91-22.45**	1.16 (.250)	1.07 (.676)	1.23 (.296)	1.28 (.265)	0.95 (.758)	0.88 (.500)	0.92 (.598)	0.84 (.305)	1.81 (.001)	1.93 (.002)	1.78 (.094)	1.96 (.096)
**22.46-23.71**	0.85 (.224)	0.77 (.104)	1.03 (.877)	1.06 (.797)	0.77 (.133)	0.63 (.022)	0.86 (.321)	0.70 (.038)	1.29 (.181)	1.33 (.193)	1.22 (.590)	1.36 (.475)
**23.72-24.80 (ref)**	1.00	1.00	1.00	1.00	1.00	1.00	1.00	1.00	1.00	1.00	1.00	1.00
**24.81-25.89**	0.91 (.466)	0.97 (.814)	0.86 (.442)	0.97 (.905)	0.92 (.578)	0.86 (.383)	0.96 (.775)	0.93 (.659)	1.48 (.026)	1.60 (.020)	1.37 (.351)	2.00 (.085)
**25.90-27.03**	0.85 (.173)	0.94 (.693)	0.77 (.168)	0.95 (.822)	0.76 (.084)	0.67 (.031)	0.84 (.218)	0.87 (.359)	1.08 (.659)	1.23 (.315)	0.83 (.620)	1.27 (.574)
**27.04-28.39**	1.05 (.697)	1.07 (.644)	0.79 (.229)	0.95 (.799)	1.00 (.989)	0.96 (.805)	1.05 (.737)	1.04 (.797)	1.55 (.012)	1.80 (.003)	0.98 (.956)	1.20 (.677)
**28.40-30.12**	1.08 (.494)	1.22 (.135)	0.87 (.442)	0.96 (.866)	1.17 (.279)	1.10 (.554)	1.27 (.065)	1.21 (.194)	1.60 (.006)	1.79 (.003)	1.76 (.075)	2.33 (.026)
**30.13-33.01**	1.34 (.011)	1.29 (.063)	1.27 (.183)	1.35 (.136)	1.25 (.144)	1.16 (.382)	1.18 (.215)	1.13 (.414)	2.11 (<.001)	2.19 (<.001)	2.35 (.006)	2.94 (.004)
**33.02+**	1.95 (<.001)	1.64 (<.001)	1.68 (.004)	1.71 (.007)	1.53 (.005)	1.16 (.400)	1.84 (<.001)	1.46 (.010)	2.66 (<.001)	2.61 (<.001)	3.07 (<.001)	2.69 (.009)
**smoking (overall)**		(<.001)		(<.001)		(<.001)		(<.001)		(.001)		(.004)
**non-smoker (ref)**		1.00		1.00		1.00		1.00		1.00		1.00
**ex-smoker**		1.33 (.001)		1.19 (.148)		1.32 (.008)		1.35 (.001)		1.11 (.301)		1.21 (.354)
**smoker**		1.93 (<.001)		1.73 (<.001)		1.90 (<.001)		1.85 (<.001)		1.53 (<.001)		1.96 (.002)
**alcohol consumption (overall)**		(<.001)		(.001)		(<.001)		(<.001)		(<.001)		(<.001)
**abstainer**		1.60 (<.001)		1.54 (.002)		1.90 (<.001)		1.80 (<.001)		1.50 (.001)		2.02 (<.001)
**low risk (ref)**		1.00		1.00		1.00		1.00		1.00		1.00
**risky**		0.97 (.630)		1.25 (.043)		1.12 (.230)		1.03 (.726)		0.96 (.657)		0.80 (.261)
**high risk**		1.55 (.016)		2.11 (.004)		2.44 (<.001)		1.87 (.002)		1.77 (.011)		0.86 (.760)
**physical activity (overall)**		(<.001)		(.008)		(.083)		(.020)		(.153)		(.718)
**no activity**		1.72 (<.001)		1.44 (.002)		1.13 (.246)		1.26 (.011)		1.15 (.203)		1.04 (.868)
**insufficient**		1.37 (<.001)		1.23 (.069)		1.24 (.027)		1.19 (.037)		1.90 (.065)		1.16 (.425)
**sufficient (ref)**		1.00		1.00		1.00		1.00		1.00		1.00
**Education (overall)**		(.388)		(.002)		(<.001)		(.001)		(.001)		(<.001)
**high school or less**		1.00		1.00		1.00		1.00		1.00		1.00
**"trade, cert, dipl"**		0.91 (.195)		1.04 (.713)		1.07 (.460)		0.90 (.220)		1.34 (.002)		0.82 (.325)
**degree or higher**		0.92 (.419)		1.58 (.001)		1.62 (<.001)		1.36 (.002)		1.43 (.002)		2.08 (<.001)
**income ('000 AUD)**		0.69 (<.001)		0.68 (<.001)		0.77 (<.001)		0.71 (<.001)		0.85 (<.001)		0.55 (<.001)
**N**	18956	14882	10576	9227	18995	14907	18995	14907	18995	14907	18995	14907

The greatest difference between genders was in stress-related mental ill-health problems. For men, both low and high BMI-deciles in model 2 were at increased odds of having a mental ill-health condition while in women there was no significant association with BMI. Of note was that overweight and obese women and obese men have a greater risk of being distressed (model 1). However, model 2 showed that men in the highest BMI-decile and women in the highest three BMI-deciles were still at significantly elevated risk of having mental ill-health conditions. A significantly elevated risk of depression was found for women in the top three deciles (this was apparent only the top decile for men).

The second series of multivariate models (model 2) which included smoking, alcohol consumption, physical inactivity and SES (Tables 2 and 3), as well as BMI-decile and age, had little impact on the relationship between BMI-decile and mental ill-health conditions although these factors had associations of their own with mental ill-health, particularly in males. In men all mental ill-health conditions were associated with an elevated odds of being a current smoker and, often, an ex-smoker. Both alcohol abstainers and those with high-alcohol-risk consumption were also consistently associated with mental ill-health conditions. Physical inactivity was inconsistently associated with mental ill-health conditions with the "insufficient" category most likely to produce a statistically significant association and a dose-response relationship was not always apparent. Increasing income greatly decreased the odds of reporting a mental ill-health problem. Education showed a similar pattern although the pattern was not as consistent across all the mental ill-health conditions.

For model 2, women showed fewer associations between the other factors and mental ill-health problems. For alcohol consumption, abstainers were at increased odds for only distress, suicidal ideation and anxiety. Similar to men, women with mental ill-health problems were also more likely to be current or ex-smokers. Physical inactivity was more likely to show a dose-response relationship with odds of mental ill-health conditions but significant associations with specific mental ill-health conditions were rare. As in men, increasing education and income were associated with a decreased odds of reporting mental ill-health problems.

## Discussion

This report is one of few to investigate the shape of the relationship between BMI and mental ill-health conditions. Non-linear associations were observed between BMI-decile and mental ill-health conditions in men and women living in South Australia. In contrast to some published research [20,28,37], greater odds of mental ill-health problems were observed only in the obese and not in the lowest BMI-decile after controlling for age, health-adverse behaviours and SES. Elevated odds were apparent for middle-aged persons, whereas younger and older individuals had a significantly lower odds of having a mental ill-health condition.

The addition to the model of health-adverse behaviours which are often associated with mental ill-health problems essentially made no change to the age and BMI associations found in the base models. As in other reports on mental ill-health, those with mental ill-health problems were more likely to smoke, and to be inactive. The relationship with alcohol was more complex with abstainers being at increased risk of mental ill-health problems while high risk consumption of alcohol was a male, rather than female, behaviour associated with the mental ill-health conditions. Higher SES seemed to be protective against the development of mental ill-health conditions, consistent with published literature.

The results of this study do not support the assertion that a greater risk of mortality in the underweight can be explained, at least in part, by an increased rate of mental ill-health conditions. These results also contradict our previous findings in a UK cohort [1] and a previous study that used an older (and much smaller) set of the SAMSS data [38] as well as two recent publications on European populations [30,31]. Study design differences may explain these discrepancies. First, the English study had only a single measure of mental ill-health which asked people if a doctor had told them they had "a mental ill-health problem". This is a generic term which would encompass everything from mild distress to major debilitating mental ill-health disorders. One of the European studies [30] was a psychiatric morbidity survey which used the Clinical Interview Schedule(CIS) which is a robust measure of mental ill-health. Unfortunately the CIS process is considered too long for a health survey like the one used for this analysis. The current study allowed for assessing mental ill-health via the Kessler Distress Scale, suicidal ideation and self-reports of three specific mental ill-health conditions plus a summary variable. Only the distress scale had any increased odds in the underweight suggesting that the underweight do not suffer from major mental ill-health problems but are probably less satisfied with life. This could be tested with survey data that has more specific mental ill-health instruments.

Another difference is that we elected to look at the distribution of mental ill-health conditions across BMI and so categorized BMI into deciles so that we would have a large sample size in the smallest category and an opportunity to examine the shape of the relationship and clearly identify the nadir of the curvilinear relationship. The Whitehall Study group has tested several methods for examining the shape of the BMI and mortality relationship [39] and recommended using both a linear and quadratic term in analysis and both recent European studies used non-linear techniques [30,31]. Because there was not sufficient published information of the potential shape of the relationships under study in this project the decision was made to go with the decile approach which would allow the shape to be examined. The problem is that while giving us 10 reasonably even-sized categories, the deciles do not strictly stick to the WHO recommended category cut-offs which makes comparison with other studies difficult. We do note that it is not uncommon for researchers to use a lower cut-off of 20 for the acceptable weight category (eg. [13]), presumably to increase the number of participants in the underweight category. We had similar problems even given the large size of this dataset and people with a BMI of less than 18.5 made up 20% of the lowest decile while those with a BMI of less than 20 made up 60%. This highlights the problem of studying the lower end of the BMI spectrum in some populations where few people have a low BMI. But it is important to note that self-report measures of height and weight are extremely inaccurate in the underweight. A recent publication reporting that about one third of those classified as underweight from measured parameters were misclassified as normal category using self reports [40]. Our lowest decile might therefore be expected to include many of these misclassified people.

What the use of deciles does make clear is the non-linear relationship between BMI and mental ill-health and health behaviours. Treating BMI as a continuous variable in analysis clearly violates the primary assumption of linearity between the explanatory and outcome variables in linear regression. But this analysis also demonstrates that the conventional BMI categories may not reflect the biologic reality because for some mental ill-health measures the lowest odds were in the BMI-decile that fell just above the acceptable weight-overweight cut-off in the WHO categories.

This study had the advantage of having a large number of participants drawn from the general population so that the conclusions are likely to be generalizable to the South Australian population. The disadvantages are the self reports of height, weight and mental ill-health conditions. But, the underweight are most likely to over-report their weight [41] leading to them being misclassified upward (eg. [42]) and so reducing the possibility of our finding a relationship when there is one. The self-reported mental ill-health condition may also be under-estimated in this study because it is perceived as a socially undesirable condition [43]. Measures of medication use, use of mental health services or possible hospitalization for mental ill-health would have provided more objective measures of mental ill-health status. In addition, the planners/designers of general health surveys need to start including more robust and validated measures of mental ill-health although we recognize that the problems of survey length often preclude the use of some of the best validated measures. Even given the problem of length many surveys emphasize physical rather than mental health. But, given the rate and economic burden of, for example, depression in the Australian population (http://www.upliftprogram.com/depression_stats.html) this failure to investigate mental ill-health is perhaps an unwise decision.

Using the telephone to conduct these interviews and the electronic white pages as the sampling frame can be seen as a limitation of the study and could potentially produce biased estimates because it excludes people who do not have a landline telephone connected or are not listed in the White Pages (including the homeless, those living in sheltered accommodation or nursing homes) [44]. It is possible that some of these groups are more likely to have a mental ill-health condition, therefore, the prevalence estimates could be an underestimation. It may also be that the mentally ill are more likely to be non-responders although the response rates from SAMSS could be considered moderately acceptable for a population survey of this kind.

The strength of this study lies in the large, representative sample size and the inclusion of a broader array of mental ill-health measures, health-related risk factors and socio-demographic variables. However, SAMSS is a surveillance system which is limited to key national, state and regional indicators and contains broad questions rather than an in-depth investigation of one particular topic.

## Conclusion

In conclusion, this study has provided no support for the hypothesis of increased mental ill-health problems in the underweight. It has demonstrated the non-linear relationships between BMI and mental ill-health and between BMI and health-adverse behaviours.

## Competing interests

The authors declare that they have no competing interests.

## Authors' contributions

All authors contributing to the conceptual design of the projects, and read and commented on drafts of the paper. SJK conducted the analyses with the collaboration of EDG. All authors read and approved the final manuscript.

## Pre-publication history

The pre-publication history for this paper can be accessed here:

http://www.biomedcentral.com/1471-2458/11/765/prepub
